# Belimumab for Immune-Mediated Necrotizing Myopathy Associated With Anti-SRP Antibodies: A Case Report and Retrospective Review of Patients Treated With Anti-B-Cell Therapy in a Single Center and Literature

**DOI:** 10.3389/fimmu.2021.777502

**Published:** 2021-12-02

**Authors:** Bei-Bei Cui, Yun-Ru Tian, Xin-Yue Ma, Geng Yin, Qibing Xie

**Affiliations:** ^1^ Department of Rheumatology and Immunology, West China Hospital, Sichuan University, Chengdu, China; ^2^ West China School of Medicine, West China Hospital, Sichuan University, Chengdu, China

**Keywords:** immune-mediated necrotizing myopathy, SRP antibody, refractory IMNM, belimumab, BAFF, rituximab

## Abstract

**Background:**

Immune-mediated necrotizing myopathy (IMNM) is characterized by markedly elevated creatinine kinase and histologically scattered necrotic muscle fibers and generally associated with autoantibodies against signal recognition particle (SRP) or 3-hydroxy-3-methylglutaryl-coA-reductase (HMGCR). Poor clinical response to conventional therapies and relapses commonly occur in severe cases. Anti-B-cell therapies have been used in refractory/relapsing cases.

**Methods:**

The characteristics of a patient with IMNM associated with anti-SRP antibodies including physical examination, laboratory tests, and disease activity assessment were evaluated. Conventional therapy, belimumab treatment schedule, and follow-up data were recorded. Medical records of IMNM patients treated in our department from September 2014 to June 2021 were reviewed to evaluate the efficacy and safety of anti-B-cell therapy for anti-SRP IMNM. A literature review of patients with anti-SRP IMNM treated with anti-B-cell therapies was performed.

**Results:**

We describe a case of a 47-year-old woman with IMNM associated with anti-SRP antibodies who relapsed twice after conventional therapy but showed good response and tolerance to belimumab at 28 weeks follow-up. In this review, three patients from our department were treated with rituximab. Two of the three patients rapidly improved after treatment. Twenty patients and five retrospective studies were included in the literature review. All patients were administered rituximab as an anti-B-cell drug.

**Conclusion:**

Despite a lack of rigorous clinical trials, considerable experience demonstrated that anti-B-cell therapy might be effective for patients with IMNM associated with anti-SRP antibodies. Belimumab in association with steroids might be an encouraging option for refractory/relapsing cases.

## Introduction

Immune-mediated necrotizing myopathy (IMNM), also known as necrotizing autoimmune myopathy, is characterized by markedly elevated creatinine kinase and histologically scattered necrotic muscle fibers and generally associated with autoantibodies against signal recognition particle (SRP) or 3-hydroxy-3-methylglutaryl-coA-reductase (HMGCR) (1). Poor clinical response to conventional therapies and relapses commonly occur in severe cases.

In previous reports, anti-B-cell therapy, especially rituximab (RTX), an anti-monoclonal CD20 antibody, has been used in IMNM (2–12). In some cases, patients benefited from RTX, while in other cases, patients showed poor response or died from complications, such as infection (2–12).

Belimumab is a human monoclonal antibody targeting B-cell-activating factor (BAFF). Belimumab has been used in several rheumatoid diseases, including systemic lupus erythematosus, Sjogren’s syndrome, systemic sclerosis, and antiphospholipid syndrome (13–16).

In this study, we report a case of a patient with anti-SRP IMNM who relapsed twice after conventional therapy but showed a good response and tolerance to belimumab. We also reviewed patients with anti-SRP IMNM who received anti-B-cell therapy in our department and in the literature.

## Patients and Methods

### Case Record

Patient characteristics, including medical history, physical examination, laboratory tests, and radiological examinations, were recorded. Disease activity was assessed using the Myositis Disease Activity Assessment Visual Analogue Scale (MYOACT), Myositis Intention-to-Treat Activity Index (MITAX), 36-item Short Form Health Survey Physical Component Score (SF-36 PCS), 36-item Short Form Health Survey Mental Component Score (SF-36 MCS), and Functional Assessment of Chronic Illness Therapy-Fatigue (FACIT-F). Conventional therapy, belimumab treatment schedule, and follow-up data were recorded.

### Retrospective Review of Patients With Anti-SRP IMNM Treated With Anti-B-Cell Therapies at a Single Center

We retrospectively reviewed all the medical records of patients in our institution between September 2014 and June 2021. Patients treated with anti-B-cell therapy for anti-SRP IMNM were included. All the subjects meet the 119th ENMC or 224th ENMC classification criteria for IMNM (1, 17).

Clinical characteristics, treatment schedules, and follow-up data were recorded.

Refractory was defined as disease worsening after treatment with high-dose glucocorticoids (equivalent of prednisone 1.0 mg/kg/day for at least 1 month) and at least one immunosuppressant (including methotrexate, azathioprine, and mycophenolate mofetil) or intravenous immunoglobulin.

### A Literature Review of Patients With Anti-SRP IMNM Treated With Anti-B-Cell Therapy

We searched in PubMed, Web of Science, Embase, and Cochrane for all cases of anti-SRP IMNM treated with anti-B-cell therapy, until June 2021. All items of anti-B-cell agents that have been presented in Cochrane were included in the study; these include the following: RTX, rituxan, mabthera, ofatumumab, GA101, ofatumumab, inotuzumab, SM03, epratuzumab, belimumab, LY2127399, imalumab, VAY736, tabalumab, AMBER, isatuximab, SAR650984, daratumumab, dara, or MOR202. The disease was searched with “exp Neuromuscular Disease/or (neuromuscular disease or neuromuscular disorder or muscular disease or muscular disorder or muscle disease).tw. or exp Muscular Disease/or exp Myositis/or (myotoni dystroph, myotoni disorder, muscular dystroph, myopath, myotonia congenita, or paramyotonia congenita).tw. or (periodic paralysis or central core disease or mitochondrial cytopath).mp. or glycogen storage disease, glycogen storage disorder, fatty oxidation disorder, inflammatory myopathy, polymyositis, dermatomyositis, inclusion body myositis, or endocrine myopathy).mp.” and “anti-srp.mp. or anti-signal recognition particle. or signal recognition particle.mp.”

Inclusion criteria are as follows: (1) adults >18 years of age, (2) following the 119th ENMC or 224th ENMC classification criteria for IMNM, and (3) the patient tested positive for anti-SRP antibodies. Patients with other myopathy diseases were excluded from the study.

## Results

### Belimumab Treatment in a Patient With Relapsing Anti-SRP IMNM

A 47-year-old woman presented with upper and lower extremity weakness. Elevated creatinine kinase (CK) and positive antinuclear antibodies (ANA) and anti-SRP antibodies were identified. Other autoantibodies, including anti-Sm, anti-RNP, anti-SSA/Ro, anti-SSB/La, anti-topoisomerase 1, anti-hystidyl-tRNA synthetase, anti-ribosomal P, and anti-chromatin, were negative. A muscle biopsy showed scattered necrotic muscle fibers. The patient was diagnosed with immune-mediated necrotizing myopathy and she began to receive prednisone at a dose of 50 mg/day and methotrexate at a dose of 15 mg once weekly. The patient responded well to the treatment, and the dose of prednisone was gradually tapered to 10 mg/day in 1 year.

Seventeen months later, muscle weakness recurred and creatinine kinase increased again. The patient was administered cyclosporine 75 mg twice daily, combined with methotrexate and prednisone. Creatinine kinase decreased but did not return to the normal range, and muscle weakness persisted. The patient was hospitalized for a second relapse of the disease 7 months later. Belimumab was added at a dose of 10 mg/kg once every 2 weeks for 6 weeks, followed by 10 mg/kg once a month. Meanwhile, the dose of prednisone was changed to 60 mg once a day as well as methotrexate at a dose of 12.5 mg once a week. The patient showed a good response and tolerance to this combination therapy, and no adverse effects were noted with the use of belimumab. All scores, including MYOACT, MITAX, SF-36 PCS, SF-36 MCS, and FACIT-F, improved after belimumab therapy (**Table 1**). Twenty-three weeks later, the CK level of the patient decreased to normal and was maintained while the dose of prednisone was gradually tapered to 12.5 mg once a day. The anti-SRP antibody test results were negative.

**Table 1:** Biochemical variables and scale scores in response to different methods of immunosuppression.

	Onset time of treatment	First flare of the disease	Second flare of the disease	Onset time of belimumab	2 Weeks after belimumab treatment	5 Weeks after belimumab treatment	13 Weeks after belimumab treatment	23 Weeks after belimumab treatment	28 Weeks after belimumab treatment
	December 12, 2017	March 18, 2019	November 28, 2019	January 04, 2020	January 21, 2020	February 10, 2020	April 06, 2020	June 15, 2020	July 20, 2020
	MTX, predinisone	MTX, predinisone, cyclosporine		MTX, predinisone, belimumab					
Biochemical variable^a^									
Creatinine kinase (IU/L)	1,529^b^	1,417^c^	4,850	1,073	1,544	1,446	545	131	119
LDH (IU/L)	395	439	613	493	509	537	494	229	213
HBDH (IU/L)	316	378	492	413	445	435	432	190	177
Count of B cells (cell/µl)				534				438	
ANA	1:10,000			1:3,200				1:1,000	
Anti-SRP	+							Negative	
**Disease activity by scale scores**									
MYOACT				6.2/60				2.1/60	1.9/60
MITAX				9/63				5/63	5/63
MDI-Muscle Severity				6.6/110				1.8/110	1.3/110
MDI-Muscle Extent				1/38				1/38	1/38
**Health-related quality of life (mean (SD) g)**									
SF36-MCS				81.1				86.6	86.6
SF36-PCS				44.8				61.8	61.8
FACIT-F				14				12	12
HAQ				0.4				0.1	0.1

MTX, methotrexate; LDH, lactate dehydrogenase; HBDH, hydroxybutyrate dehydrogenase; MYOACT, Myositis Disease Activity Assessment Visual Analog Scale; MITAX, Myositis Intention-to-Treat Activity Index; MDI, Myositis Damage Index; SF-36 PCS, 36-item Short-Form Health Survey Physical Component Score; SF-36 MCS, 36-item Short-Form Health Survey Mental Component Score; FACIT-F, The Functional Assessment of Chronic Illness Therapy-Fatigue; HAQ, Health Assessment Questionnaire.

a The reference ranges for the biochemical variables are as follows: for creatinine kinase, 19 to 226 IU/L; for LDH, 120 to 250 IU/L; for HBDH, 72 to 182 IU/L; and for count of B cell, 175–332 cells/μl.

b After treatment, strength and creatine kinase returned to normalization.

c After treatment, creatinine kinase decreased but not back to normal range and muscle weakness persisted.

### Retrospective Review of Patients With Anti-SRP IMNM Treated With Anti-B-Cell Therapies in a Single Center

A total of 112 patients with anti-SRP IMNM who visited our department between September 2014 and June 2021 were reviewed. Only three patients were treated with RTX (anti-B-cell therapy). The first patient was a refractory case and received RTX six times (a dose of 500 mg/week for 2 weeks, then repeated 1 month later and 500 mg, two times, 1 year apart). The symptoms persisted. The other two patients responded to RTX; however, herpes zoster developed in the third patient after the first infusion and the treatment was discontinued. Patients’ characteristics, laboratory data, treatment schedules, and outcomes are presented in **Table 2**.

**Table 2:** Retrospective review of patients with anti-SRP IMNM treated with anti-B-cell therapies in a single center.

Patient No./Age/Gender	Severe symptoms	Strength prior to RTX^a^	Strength after RTX^a^	CK prior to RTX (IU/L)	CK after RTX (IU/L)	Other outcomes	RTX treatment schedule	Cointerventions	Adverse event
1/40/M	None	2/5	2/5	1,851	1,014	None	2 doses of 500 mg/weekly, repeated 1 month later	CsA, Pred	None
2/57/M	Dysphagia, cardiomyopathy	3/5	5/5	5,811	390	Improvement of myocardial markers	2 doses of 100 mg/weekly, and 2 doses of 500/weekly 1 month later	Pred, CTX	None
3/54/M	Dysphagia, cardiomyopathy	4/5	4/5	7,238	134	Improvement of myocardial markers	1 dose of 100 mg	MMF, IVIG	Herpes zoster infection

MTX, methotrexate; IVIG, intravenous immunoglobulin; CTX, cyclophosphamide; MMF, mycophenolate mofetil; CsA, cyclosporine A; Pred, prednisone; RTX, rituximab.

a Strength was evaluated with MRC score.

### A Literature Review of Patients With Anti-SRP IMNM Treated With Anti-B-Cell Therapy

A total of 124 articles were identified from the database. After excluding articles that are not written in English or patients who matched the exclusion criteria, 15 articles were finally selected. Twenty patients with anti-SRP IMNM and RTX treatment from case reports and case series were reviewed, and five retrospective studies were included. Details are summarized in **Tables 3**, **4**.

**Table 3:** Case reports and case series of anti-SRP IMNM patients treated with anti-B-cell therapies.

Study (year)	Age/Gender	Severe symptoms	Prior treatment	RTX treatment schedule	Cointervention	Outcome	Adverse effects
Mazeda et al. (2021) (2)	76/F	Dysphagia	Pred, IVIG	One treatment A		Rapid symptomatic improvement	N/A
Ying et al. (2020) (3)	34/F	EN, dysphagia	MP	100 mg on Day 0, then 500 mg on Day1	MP	Decline in CK and improvement in strength	N/A
Mehta et al. (2019) (4)	30/F	15-week gestation	MP, IVIG,AZA, RTX repeated every 6 months until pregnancy	One infusion	Pred	Decline in CK and improvement in strength	N/A
Novoa Medina et al. (2018) (5)	30/F		CS, IVIG, AZA	1 treatment, repeated 6 months later	MTX	Clinical remission, but relapsed with MTX tapering	N/A
Komiya et al. (2018) (6)	71/M	Dysphagia, lymphoma	CS, IVIG, tacrolimus	R-CHOP therapy every 3 weeks for 6 cycles and an additional 2 cycles	Pred, CTX, DEX, VCR	Complete remission	N/A
Mamarabadi et al. (2018) (7)	28/F	Dysphagia	CS, IVIG	One treatment B for 5 times, repeated every 6 months	CS, IVIG	Decline in CK and improvement in strength	N/A
Valiyil et al. (2010) (8)	20/F		CS, AZA, MTX	1 treatment A	MTX and Pred	Decline in CK and improvement in strength	N/A
	34/F	Dysphagia	Pred, MTX, AZA, IVIG	1 treatment A	PE 5 times	Decline in CK and improvement in strength	N/A
	44/F		CS, MTX, MMF	1 treatment A, repeated 6 months later and 1 infusion 8 months later	CS	Decline in CK and improvement in strength	Facial abscess 1 month after initial dosing
	72/M	Dysphagia	CS, IVIG, PE	1 infusion		Decline in CK	Pneumonia and congestive heart failure, died 1 month later
	21/F		CS, MTX	One treatment A		Decline in CK and improvement in strength	Herpes zoster infection 3 months later
	26/F	Dysphagia	CS, IVIG, MTX, and MMF	1 treatment A		Decline in CK and improvement in strength	N/A
	51/M		Pred, MTX, MMF	1 treatment A		Decline in CK	N/A
	32/F		Pred, AZA, MTX, IVIG	1 treatment A		Decline in CK and improvement in strength	N/A
Fernandes das Neves et al. (2015) (9)	50/F	Dyspnea	MTX, CTX, IVIG, Pred.	1 treatment A, repeated every 6 months	CTX, Pred	Clinical remission	N/A
Curtin (2016) (10)	54/M		CS, IVIG	1 treatment A	CTX, Pred	Decline in CK and improvement in strength	N/A
Whelan and Isenberg (2009) (11)	44/F		CS, AZA, MTX	1 treatment A	MP and CTX	Decline in CK, relapsed 3 months later	Herpes zoster infection
	41/F		AZA, MTX, IVIG, MMF	1 treatment A		Decline in CK and improvement in strength	N/A
Arlet (2006) (12)	20/M		CS, IVIG, CS,PE, CsA, CTX, MMF	1 treatment B for 4 times, repeated every 4 months for 3 times	Pred, PE	Symptomatic improvement, but then relapsed 6 months after second infusion	A flare of hepatitis B with delta coinfection after the 2nd single additional infusion
	24/F		Pred, IVIG, MTX, AZA, PE, CTX	1 treatment B for 4 times and every 4 months	Pred	Decline in CK and improvement in strength	N/A

EN, erythema nodosum; AZA, azathioprine; CK, creatinine kinase; CTX, cyclophosphamide; IMNM, immune-mediated necrotizing myopathy; IVIG, intravenous immunoglobulin; MTX, methotrexate; N/A, information not available; PE, plasma exchange; RTX, rituximab; Pred, prednisone; CsA, cyclosporine A; CS, glucocorticoid; DEX, dexamethasone; MMF, mycophenolate mofetil.

Treatment A protocol: two doses of 1,000 mg, 2 weeks apart. Treatment B protocol: one dose of 375 mg/m^2^ weekly. One infusion: one dose of 1,000 mg.

**Table 4:** Literature review of studies on anti-SRP IMNM patients treated with anti-B-cell therapies.

Study (year)	Population	No. of anti-SRP IMNM patients treated with RTX	Study design	RTX schedule	Outcome
Benveniste et al. (2011) (18)	8 anti-SRP IMNM/PM	4/8	R	Not specified	3 patients significantly improved in strength, 1 patient slightly improved in strength.
Pinal-Fernandez (2017) (19)	37 anti-SRP IMNM	21/37	R	Not specified	13 patients responded^b^; 4 patients could not be evaluated.
Needham (2016) (20)	20 IMNM (2 anti-SRP IMNM)	1/2	R	Not specified	The patient responded very well but relapsed with prednisone weaning.
Allenbach et al. (2018) (1)	18 IMNM	18 IMNM^a^	R	1 g D1 and D14 followed by a median of 4 infusions (1 g each, ranging from 1 to 10)	Remission was obtained in 9 patients.
De Visser (2019) (21)	64 IMNM (15 with anti-SRP antibodies)	3 IMNM^a^	R	Not specified	N/A

R, retrospective study.

a Not specified for anti-SRP IMNM.

b Response is defined as strength increased 2 points or CK levels declined by 10-fold within 6 months.

## Discussion

To our knowledge, this is the first case of belimumab in anti-SRP IMNM. The patient showed good response and tolerance to belimumab.

There are still no randomized trials or large enough case series to make formal recommendations for IMNM treatment. Based on the European Neuromuscular Center workshop, corticosteroids are considered the first-line treatment (1). High-dose corticosteroids should be used immediately upon diagnosis. For patients with an incomplete response to corticosteroid monotherapy or multisystem involvement, second-line treatments are warranted; these include methotrexate, azathioprine, and mycophenolate mofetil. In some cases, cyclosporine and tacrolimus may be used as adjuncts. In addition to conventional immunosuppression, IVIg is considered an effective treatment for initial therapy, especially in anti-HMGCR myopathy (1).

In our study, we reviewed all B-cell therapies for IMNM, and only RTX was identified to be effective (1–12, 18–21). B-cell depletion therapy with RTX in anti-SRP IMNM is commonly effective. As our study showed, all patients from case reports and case series showed a decline in CK, while three patients relapsed during tapering. In addition, five patients developed infections after RTX therapy, and one patient died from pneumonia and congestive heart failure. From the reported literature, it was gathered that most patients responded; in one study, however, only half of patients achieved remission (1). The author of the study supposed that a low ratio of remission might be related to the delay in RTX use. Despite a lack of rigorous clinical trials, considerable experience has demonstrated that anti-B-cell therapy might be effective for patients with IMNM.

Although belimumab has never been reported for use in IMNM treatment, the important role of BAFF or B-lymphocyte stimulator in the pathogenesis of idiopathic inflammatory myopathies (IIM) has been demonstrated in previous studies (22–24). In a study by Yuan, 10 of 29 patients with refractory anti-SRP IMNM showed positive BAFF in necrotic tissue regenerated muscle fibers and individual lymphocytes, while BAFF receptor was found in 24 of 29 patients. Moreover, refractory patients with anti-SRP IMNM had more BAFF receptors than nonrefractory patients. These findings suggest that BAFF and its receptors may participate in muscle fiber injury (22).

The efficacy and safety of belimumab in other autoimmune diseases have been evaluated in randomized clinical trials. The BLISS trial, a randomized, double-blind, placebo-controlled trial, demonstrated the efficacy of belimumab in SLE (13). The BLISS-LN study, a multicenter, randomized, double-blind trial included 448 patients with lupus nephritis. At week 104, primary responses occurred more often in the belimumab group than in the placebo group. Infection and infestation occurred in 15 of 224 patients in the belimumab group and 18 of 224 patients, while the number of infection-associated deaths were equal to the two groups (three patients in each group) (25). In a bicentric prospective 1-year open-label trial on Sjogren’s syndrome, patients achieved improvement in several aspects, including disease activity index, dryness, fatigue, and VAS scores. Only one of 30 patients suffered from a severe adverse event (pneumococcus meningitis) (14). A multicenter, double-blind, placebo-controlled trial on the efficacy and safety of belimumab in IIMs is ongoing by Northwell Health (NCT02347891).

Consistent with previous reports, not only improvement of disease activity but also a decline in anti-SRP antibodies was observed in our case. Some evidence has demonstrated that anti-SRP antibodies may participate in the pathogenesis of IMNM by triggering an immune reaction, resulting in the release of myotoxic cytokines (8, 22, 26). *In vitro*, positive SRP was found on the plasma membrane of cultured myoblast cells stained with anti-SRP serum (27). In animal models, muscle weakness was observed in C57/Bl6 or Rag2-deficient or complement 3-deficient mice after passive IgG transfer from patients with anti-SRP IMNM (28). Moreover, SRP protein was identified in the muscle of anti-SRP IMNM patients *via* colabeling with the transsarcolemmal protein dysferlin and sarcoplasmic neural cell adhesion molecule, respectively, and further cellular experiments demonstrated exposed SRP protein localized at the surface of myotubes (29).

There are some limitations to this case: since this is the first case describing belimumab in IMNM, more cases and studies are needed to confirm the effects. Based on this case, we observed that belimumab was effective in IMNM associated with anti-SRP antibodies and suggest that belimumab might be option for severe cases.

## Conclusion

Belimumab improved the clinical condition of our patient without any severe adverse events. In the review of the records of our center and literature, B-cell therapy with RTX benefited some patients with anti-SRP IMNM, but at the same time, increased the risk of infection. In conclusion, the present study demonstrates that belimumab in association with steroids might be an encouraging option for refractory/relapsing cases.

## Data Availability Statement

The original contributions presented in the study are included in the article/supplementary material. Further inquiries can be directed to the corresponding author.

## Ethics Statement

The studies involving human participants were reviewed and approved by the ethics committee of West China Hospital. The patients/participants provided their written informed consent to participate in this study. Written informed consent was obtained from the individual(s) for the publication of any potentially identifiable images or data included in this article.

## Author Contributions

All authors contributed to one or more of the following aspects of the manuscript: conception, acquisition of data, drafting, and revising the article. All authors contributed to the article and approved the submitted version.

## Funding

This work was supported by the Clinical Research Incubation Project, West China Hospital, Sichuan University (2019HXFH038).

## Conflict of Interest

The authors declare that the research was conducted in the absence of any commercial or financial relationships that could be construed as a potential conflict of interest.

## Publisher’s Note

All claims expressed in this article are solely those of the authors and do not necessarily represent those of their affiliated organizations, or those of the publisher, the editors and the reviewers. Any product that may be evaluated in this article, or claim that may be made by its manufacturer, is not guaranteed or endorsed by the publisher.

## Abbreviations

IMNM, immune-mediated necrotizing myopathy; SRP, signal recognition particle; HMGCR, 3-hydroxy-3-methylglutaryl-coA-reductase; RTX, rituximab; BAFF, B-cell-activating factor; IIM, idiopathic inflammatory myopathies.
